# Bmp and Shh Signaling Mediate the Expression of *satb2* in the Pharyngeal Arches

**DOI:** 10.1371/journal.pone.0059533

**Published:** 2013-03-21

**Authors:** Kelly Sheehan-Rooney, Mary E. Swartz, C. Ben Lovely, Michael J. Dixon, Johann K. Eberhart

**Affiliations:** 1 Section of Molecular, Cell and Developmental Biology, Institute of Cellular and Molecular Biology, University of Texas at Austin, Austin, Texas, United States of America; 2 Faculty of Life Sciences and Faculty of Medical and Human Sciences, Manchester Academic Health Sciences Centre, Michael Smith Building, University of Manchester, Manchester, United Kingdom; Texas A&M University, United States of America

## Abstract

In human, mutation of the transcription factor *SATB2* causes severe defects to the palate and jaw. The expression and sequence of SATB2 is highly conserved across vertebrate species, including zebrafish. We sought to understand the regulation of *satb2* using the zebrafish model system. Due to the normal expression domains of *satb2*, we analyzed *satb2* expression in mutants with disrupted Hh signaling or defective ventral patterning. While *satb2* expression appears independent of Edn1 signaling, appropriate expression requires Shha, Smo, Smad5 and Hand2 function. Transplantation experiments show that neural crest cells receive both Bmp and Hh signaling to induce *satb2* expression. Dorsomorphin- and cyclopamine-mediated inhibition of Bmp and Hh signaling, respectively, suggests that proper *satb2* expression requires a relatively earlier Bmp signal and a later Hh signal. We propose that Bmp signaling establishes competence for the neural crest to respond to Hh signaling, thus inducing *satb2* expression.

## Introduction

Cranial neural crest cells are a highly specialized vertebrate-specific cell type which give rise to diverse structures including most of the bone and cartilage of the head, face and palate. In all vertebrates, this pluripotent cell population migrates to the periphery in three streams and condenses within transient structures known as pharyngeal arches [1], [2]. In amniotes, neural crest cells of the anterior-most stream contribute to the jaw and palatal skeleton [3], [4]. The palatal precursors reside in the frontonasal prominence to generate the primary palate, and the maxillary prominence of the first arch to generate the secondary palate and upper jaw [5]. The mandibular prominence of the first arch contributes to the lower jaw. The second crest stream migrates to the second arch and contributes skeletal elements such as the hyoid bone 6,7. The third stream populates the more posterior arches. Zebrafish have similar neural crest cell populations, with frontonasal and maxillary crest populations generating distinct regions of the anterior neurocranium, or zebrafish palate [8], [9], [10], [11]. As in amniotes, maxillary and mandibular neural crest give rise to the upper and lower jaws, respectively and neural crest cells of the second arch form the dorsal and ventral jaw support elements [8], [12].

Pharyngeal arches consist of a mesodermal core surrounded by neural crest cell-derived mesenchyme that is encapsulated by epithelia (the ectoderm and the endoderm). Reciprocal interactions between these different cell types are critical to establish the signaling networks required to generate the multitude of craniofacial elements of the appropriate size and shape [5], [9], [13]. Craniofacial growth and specification is controlled by the interaction of numerous signaling pathways, including those mediated via Sonic hedgehog (Shh), Endothelin-1 (Edn1) and Bone morphogenic protein (Bmp) [5]. Across vertebrates, the Bmp and Edn1 pathways pattern of the jaw. Mice and zebrafish carrying mutations of *Edn1* show reduced expression of genes known to be essential for mandibular development such as *Hand2* [14], [15], [16], [17]. Bmp also functions in ventral patterning of the pharyngeal arches with activities both upstream and independent of Edn1 [18], [19], [20], [21]. Notch signaling acts antagonistically to these ventral signals to establish dorsal identity [22]. In addition to its role in ventral arch patterning, Bmp signals play crucial roles in the development of the palatal skeleton in amniotes and zebrafish [12], [23], [24], [25]. In its role during palatogenesis, Bmp signaling induces the expression of another critically important signaling molecule, Shh 25,26.

Shh is a member of the secreted family of Hedgehog (Hh) proteins and all hedgehog signaling is transduced through Smoothened (Smo). Shh is crucial for regulating patterning and outgrowth of the face and palate across vertebrate species [8], [10], [11], [27], [28], [29], [30]. Mutation of human *SHH* or its downstream effectors result in a variety of severe developmental disorders; those affecting the head and face include holoprosencephaly, cyclopia and hypotelorism (Belloni *et al.*, 1996; Dubourg *et al.*, 2007; Nanni *et al.*, 1999; Roessler *et al.*, 1996). Neural crest cells must receive Hh signaling for the appropriate expression of patterning genes in the developing craniofacial skeleton [11], [29]. In the developing palatal skeleton, Shh signaling plays a crucial role in outgrowth of the palatal shelves and in maintaining proliferation of the palatal mesenchyme [5], [31].

Considering the numerous transcription factors and signaling molecules involved in regulating craniofacial development it is not surprising that craniofacial defects are common among congenital birth defects. The most common craniofacial defects are cleft lip and/or palate and mandibular dysmorphogenesis [32]. The use of model organisms in dissecting the underlying molecular pathways regulating facial development and palatogenesis is imperative to our understanding of these processes in humans. Such work has been carried out to understand the role of the transcription factor SATB2. Mutation of this gene in human and mouse results in strikingly similar craniofacial defects including cleft palate and jaw abnormalities [33], [34], [35], [36], [37]. We have previously shown that the sequence identity of the SATB2 protein and expression of the transcript is highly conserved across vertebrate species including zebrafish [38].

Here, we use zebrafish to gain insight into how Satb2 integrates into the signaling cascades involved in craniofacial development. We show that *satb2* is a target of Bmp and Hh signaling. Using genetic mosaics, we find that neural crest cells must receive both signals for proper *satb2* expression. Using pharmacologic inhibition of each pathway, we find that Bmp signaling is required temporally before Hh signaling. Collectively, we provide some of the first evidence regarding the regulation of *satb2.*

## Materials and Methods

### Zebrafish Lines

The study was carried out in strict accordance with the recommendations in the Guide for the Care and Use of Laboratory Animals of the National Institutes of Health. The protocol was approved by the Institutional Animal Care and Use Committee at the University of Texas at Austin (AUP-2012-00053). Zebrafish (*Danio rerio*) embryos were raised at 28.5°C and staged as described previously [39]. Some embryos used for *in situ* hybridization analysis were incubated in embryo media containing PTU to prevent pigmentation. Cyclopamine experiments [8] were performed in the AB background or the transgenic *tg(fli1a;EGFP)^y1^* line, designated *fli1:EGFP* for clarity [40]. Dorsomorphin (DM – Cat. #3093; Tocris Bioscience, Ellisville, MO) was dissolved in dimethyl sulfoxide (DMSO) to a concentration of 10 mM and aliquoted for long-term storage at −20°C. Zebrafish embryos were treated with 10 µM DM or with 10 µM DMSO alone as a control. Dorsomorphin experiments were carried out in the transgenic *tg(BRE-AAVmlp:dmKO2)^mw40^* line, designated *BRE:mKO2* [41]. The inhibitory effects of dorsomorphin was verified by the loss of *BRE:mKO2* expression at the time embryos were fixed for *in situ* hybridization. The exact same settings were used for the collection of all *BRE:mKO2* images. *shha^t4^* [42], *smo^b577^* [43], *edn1^tf216b^* [16], *hand2^s6^* [44] and *sox32^ta56^* [45] mutant embryos were identified by morphology or genotyping as described previously. The *edn1* and *hand2* mutant embryos were kind gifts from The University of Oregon aquarium. Heterozygous carriers of the *smoothened* null allele, *smo,* and *sox32* allele [43], [46] were maintained on the *fli1:EGFP* background and are referred to as *smo^-^* and *sox32^−^* in the text for clarity. The *smad5^b1100^* [12] allele is a cysteine to thymidine mutation at nt 733 of the first coding exon of *smad5*. This mutation is predicted to generate a Proline to Serine missense mutation in the DNA binding MH1 domain of Smad5. PCR with primers jaa1 (5′ aagggcctcccacacgtcatct 3′) and yj32 (5′ ctggactttcaactcgtttgtagtgccatg 3′), followed by digestion with NcoI was used for genotyping. The mutant allele is 160 bp and the wt allele is 129 bp.

### Skeletal Analysis and in situ Hybridization

Zebrafish larvae were stained with Alcian Blue and Alizarin Red [47] and the cartilages flat-mounted at 5 days post-fertilization (dpf). The *satb2* probe used for *in situ* hybridization is described elsewhere [38].

### Cell Transplantations

Transplantation experiments were performed as described elsewhere [8]. Briefly, donor embryos were injected with Alexa-568 dextran at the one to two cell stage. At shield stage, donor and host embryos were placed on a depression slide in filter sterilized Ringers solution containing Penicillin and Streptomycin, immobilized in 4% methylcellulose. Using a pulled micropipette, approximately 20–30 cells were removed from donor embryos and transplanted to the neural crest domain [48] of host embryos. Transplanted embryos were allowed to recover for 15–20 minutes and then placed in filter sterilized embryo media containing Penicillin and Streptomycin.

## Results

### Multiple Signaling Pathways Regulate satb2 Expression

**Figure 1:**
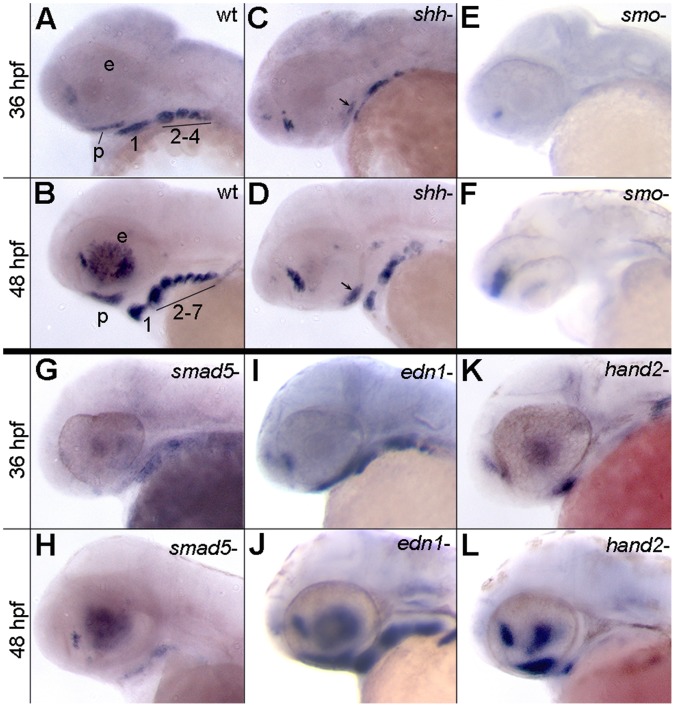
Expression of *satb2* requires Hh and Bmp signaling. (A–L) Lateral images of *satb2* expression at 36 or 48 hpf. (A–D) Compared to wild-type embryos, in 36 and 48 hpf *shha^−^* embryos, *satb2* expression is reduced in the palatal precursors (arrows in C and D) and throughout the pharyngeal arches. (E,F) Nearly all neural crest cell expression of *satb2* appears to be absent in *smo^−^* embryos. The prominent expression in panel F, is in neural tissue. (G,H) Weak to no expression of *satb2* was observed in *smad5* mutant embryos at 36 and 48 hpf. (I,J) *satb2* expression in *edn1* mutants was similar wild-type embryos at both time points analyzed. (K,L) While expression of *satb2* was absent throughout the posterior arches of *hand2* mutants, the ventral first arch and palatal precursors maintained *satb2* expression. p, palatal precursors; e, eye; The arches are numbered in A & C for clarity.

Despite its functional involvement in growth and development, the regulation of Satb2 is largely unknown. Because it plays a central role in gene regulation [49], [50], we hypothesized that Satb2 would be an effector of some of the major signaling pathways crucial for development of the face and palate. Because of the restriction of *satb2* in the ventral arch we focused our analyses on the Edn1, Bmp and Hh pathways. Expression analyses were performed at 36 and 48 hpf corresponding with the peak of *satb2* expression in crest-derived mesenchyme (Fig. 1). In zebrafish, and other vertebrates, *satb2* is expressed by neural crest cell-derived mesenchyme in both the maxillary and frontonasal prominences [33], [35], [38]. For simplicity, we refer to this expression domain collectively as palatal precursors.

Our results show that multiple signaling pathways are essential for appropriate expression of *satb2*. Hh signaling is necessary for the condensation of palatal precursors [8], [10], precluding analysis of this population. Ventral pharyngeal arch expression of *satb2* is reduced in the absence of Shha (Fig. 1 A–D) and almost completely lost in *smo* mutants, which lack all Hh signaling (Fig. 1 E,F), even though the ventral arch is present and maintains expression of other markers [11]. Bmp signaling is essential for palatogenesis and dorsal/ventral patterning of the arches in zebrafish [18], [19]. In *smad5* mutants, in which Bmp signaling is disrupted, palatal precursors have been shown to condense [12] here we show that they fail to express *satb2* (Fig. 1 G,H). Furthermore, expression of *satb2* in the ventral pharyngeal arch was greatly reduced or absent (Fig. 1 G,H). Disruption of Bmp signaling has been shown to expand dorsal arch fates at the expense of ventral fates [18], [19] strongly supporting an interpretation that these results are due to a loss of *satb2* expression as opposed to the loss of territory normally expressing *satb2*. While Edn1 signaling is also indispensible for dorsal/ventral patterning of the zebrafish pharyngeal arches [16], [51], [52], *satb2* expression appeared normal in *edn1* mutants (Fig. 1 I,J). *hand2* is a target of both Bmp and Edn1 signaling [15], [18], [19] and from our data the expression domain of *hand2* appears to overlap extensively with *satb2* [38]. Only palatal crest and a population of crest in the ventral first arch maintained *satb2* expression in *hand2* mutants (Fig. 1 K,L).

### Neural Crest Cells Must Receive both Hedgehog and Bmp Signaling for Proper satb2 Expression

**Figure 2:**
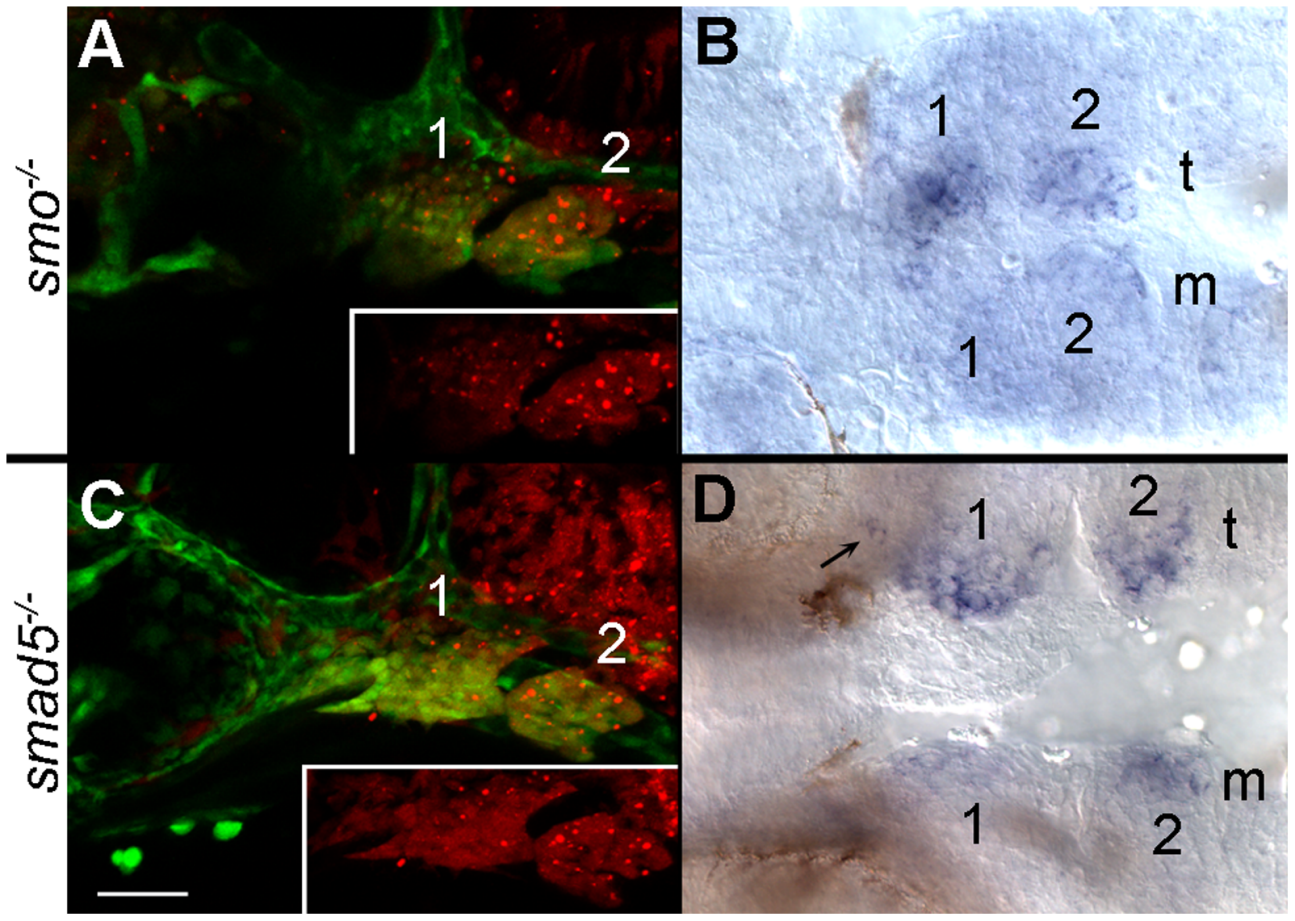
Neural crest cells require the reception of Bmp and Hh signaling to express *satb2*. (A, C) Lateral views at 36 hpf; (B, D) ventral views at 36 hpf. Both donor and host embryos were *fli1:EFGP* transgenics to allow for visualization of neural crest cells. (A) Wild-type crest transplanted into the arches of *smo* mutant embryos are shown in red in the inset. (B) *smo^+^* crest restores the ventral arch expression of *satb2* on the side of the transplant (t) as compared to the mutant side of the embryo (m). (C) Lateral views of a 36 hpf *smad5* mutant that received a transplant of *smad5^+^* neural crest. The distribution of *smad5^+^* cells (red in inset) is shown. (D) Expression of *satb2* is restored in the ventral pharyngeal arch and in the palatal precursors (arrow) on the transplanted side of the embryo. Arches 1 & 2 are numbered in each panel.

Reception of Hh signaling by neural crest cells is necessary for the proper expression of *Fox* genes and several markers of dorsal/ventral patterning [11], [29]. To determine if neural crest cells require the reception of Hh signaling for *satb2* expression, we generated genetic mosaics to restore the reception of signaling in neural crest cells. We have previously shown that an early Hh signal from the ventral brain to the oral ectoderm is required for neural crest condensation in the maxillary region [8] and thus crest transplants fail to rescue the condensation defect. Therefore, for these analyses we focused on *satb2* expression in the ventral arches. We analyzed only embryos with extensive contribution of donor neural crest cells to the ventral arches and found that transplantation of *smo^+/+^* neural crest cells into *smo* mutants rescued *satb2* expression (Fig. 2 A, B; n = 7/7). These data indicate that Hh signaling to the neural crest is necessary for *satb2* expression in the ventral arch.

The zebrafish ventral arch also responds to Bmp signaling [18], [19]. We used a similar genetic mosaic strategy to determine if neural crest cells must receive Bmp signaling to express *satb2*. Transplantation of *smad5^+/+^* neural crest cells into *smad5* mutants rescued the expression of *satb2* in the ventral pharyngeal arch (Fig. 2 C, D; n = 7/7). This transplant also sparsely populated the region of the maxillary domain expressing *satb2*. As such, a very small population of satb2 expressing cells are evident (Fig. 2, arrow). Collectively, these results demonstrate that neural crest cells must receive, at a minimum, both Hh and Bmp signaling in order to express *satb2*.

### Endoderm is a Critical Source of Signals Necessary for satb2 Expression

**Figure 3:**
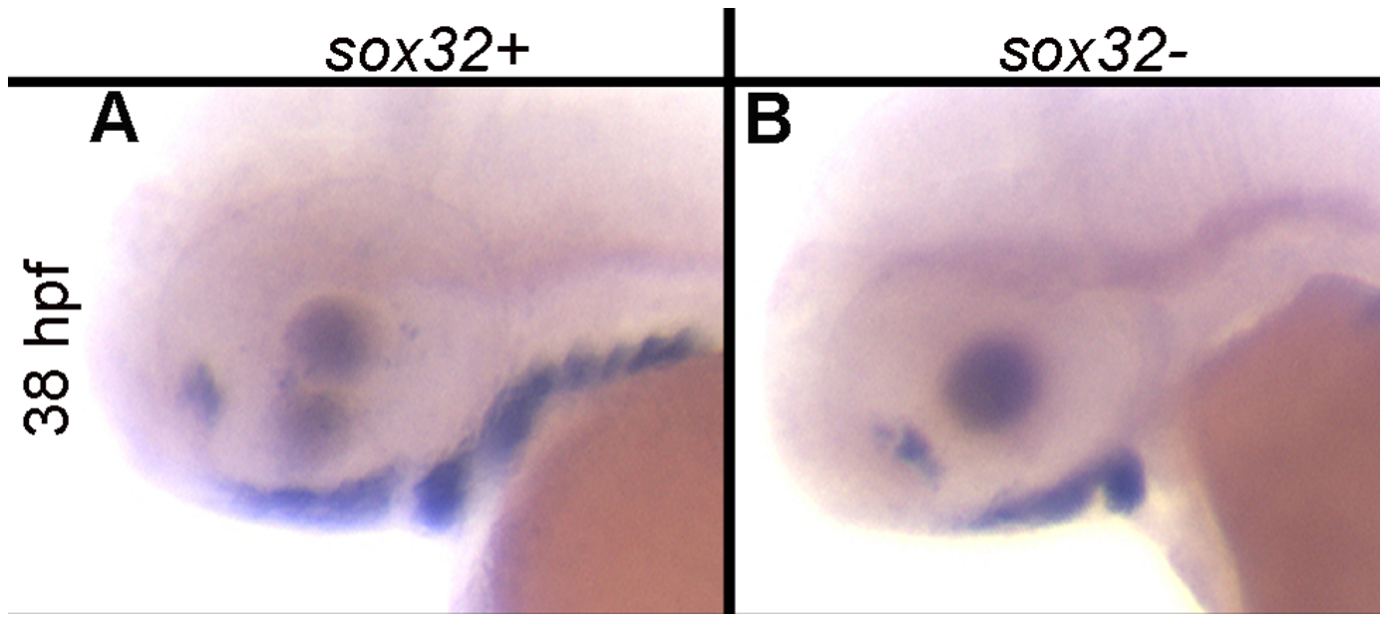
Endodermal signals are required for *satb2* expression in ventral arches 2–7. (A) At 38 hpf, *satb2* is expressed robustly in the ventral pharyngeal arches and palatal precursors of wild-type embryos. (B) Expression of *satb2* in the palatal precursors and ventral arch 1 is retained in *sox32* mutants; however, *satb2* expression is ablated in the posterior arches.

In zebrafish, *satb2* is most strongly expressed in neural crest cells in the medial region of the arch that are in close proximity to the pharyngeal endoderm, a source of both Bmp and Hh signaling [8], [12], [53], [54]. To determine if the endoderm is necessary for *satb2* expression, we analyzed *sox32* mutants, which lack endoderm [46]. At 38 hpf wild-type embryos expressed *satb2* in the ventral arch neural crest as well as palatal precursor cells (Fig. 3 A). While the expression of *satb2* was retained in the palatal precursors and the ventral first arch in *sox32* mutants, *satb2* expression was lost in the more posterior pharyngeal arches (Fig. 3 B). Collectively, these results suggest that signals from the endoderm are essential to induce *satb2* in the ventral region of the second and more posterior arches, while Bmp and Shh signaling from other tissues, such as facial ectoderm [8], [12], [53], are important to induce *satb2* in the ventral first arch and palatal precursors.

**Figure 4:**
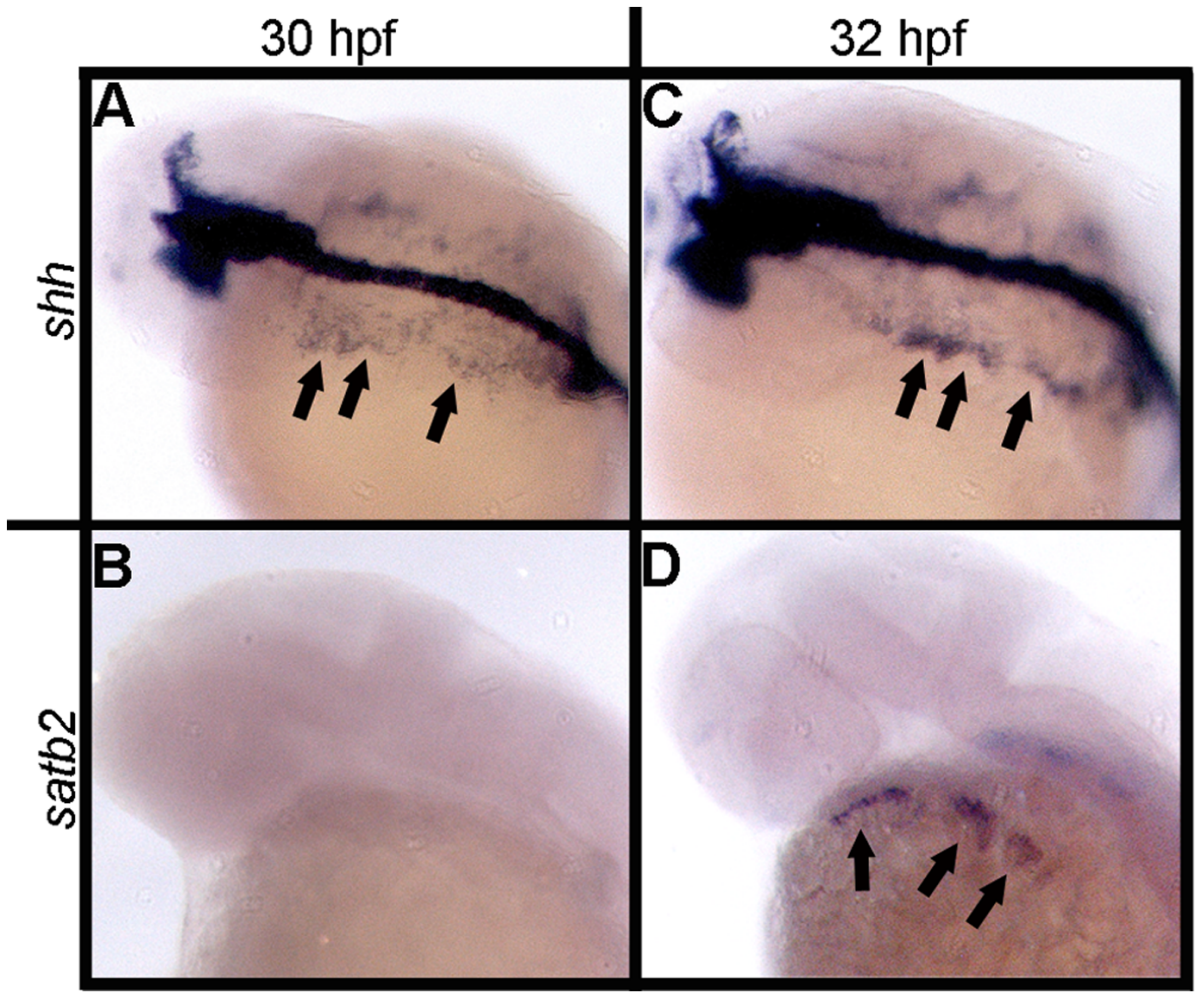
Expression of *shh* and *satb2* correlate spatially during pharyngeal arch development. (A–D) Dorsal-lateral views of 30 hpf and 32 hpf wild-type embryos. (A) *shha* expression initiates in the pharyngeal endoderm at 30 hpf while (B) *satb2* is not expressed in the craniofacial region at this time. (C, arrows) By 32 hpf *shh* expression has intensified and can be seen to arc around the presumptive arches. (D, arrows) *satb2* expression is initiated in the ventral arch by 32 hpf and lies in close proximity to the presumptive pharyngeal endoderm.

While Bmps are expressed much earlier in the endoderm [12], [54] relative to the timing of *satb2* expression in the ventral arch, the endoderm begins to express *shha* at 30 hpf (Fig. 4 A). By 32 hpf *satb2* expression is initiated in the adjacent arches 1–3 (Fig. 4 C, D). These observations suggest a model in which Bmp and Shh signaling are sequential events both necessary for the induction of *satb2* in the ventral arch. To test this model we used pharmacological inhibition of the two pathways to determine when Bmp and Shh signaling are required for *satb2* expression.

### Sequential Bmp and Shh Signaling Regulates satb2 Expression

**Figure 5:**
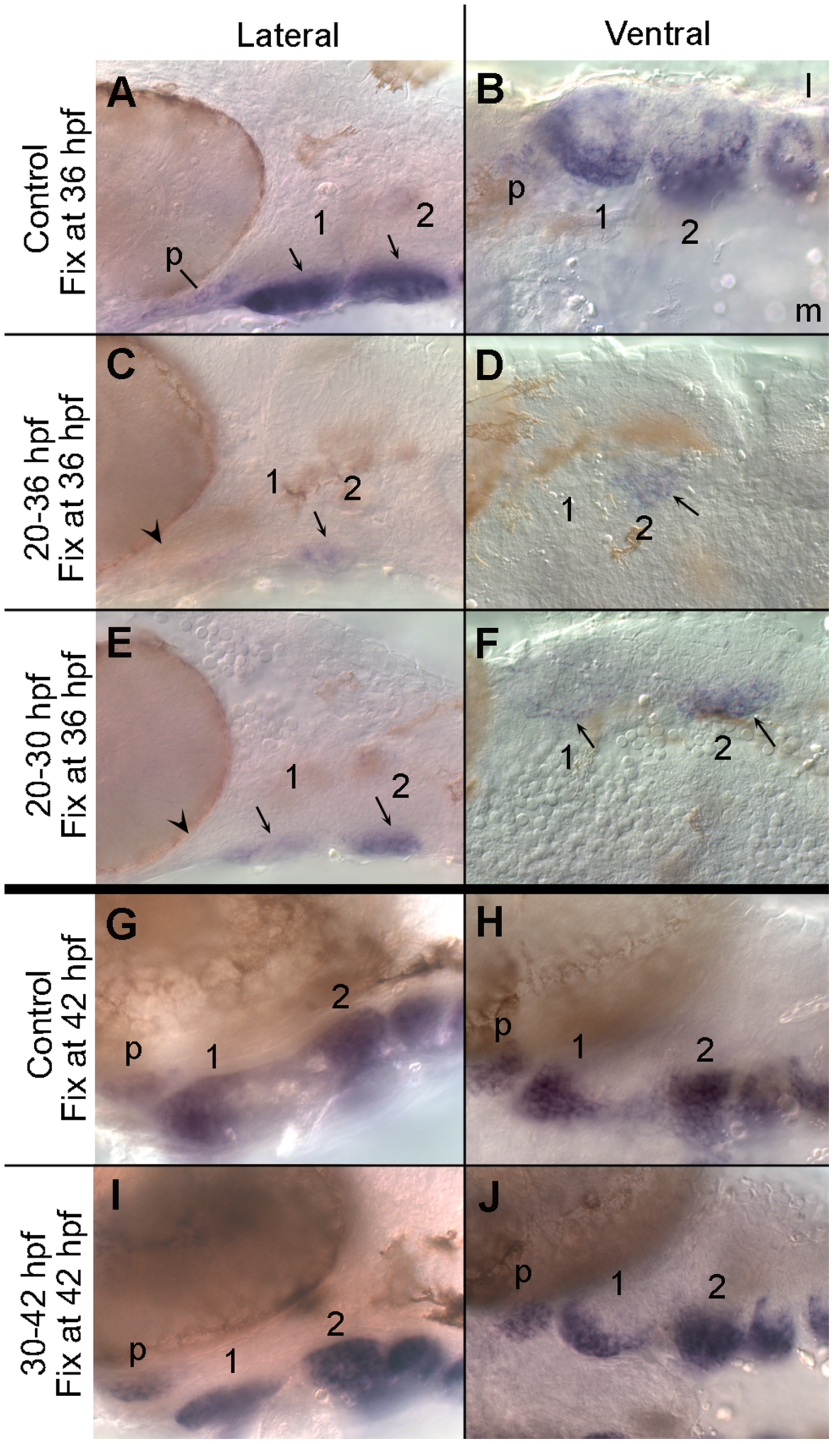
Bmp signaling is required during early arch patterning for the appropriate expression of *satb2*. (A, C, E) Lateral and (B, D, F) ventral images of 36 hpf embryos labelled with *satb2* riboprobe. (A) The ventral region of the pharyngeal arches expresses *satb2* robustly. (B) In the ventral arches, *satb2* expression is strongest medially. (C, D) Inhibiting Bmp signaling *via* dorsomorphin from 20 to 36 hpf eliminates the majority of *satb2* expression including that in the palatal precursors (arrowhead in C). Only a small population of crest in ventromedial arch 2 expresses *satb2* following this treatment (arrows in C & D). (E, F) When Bmp signaling is blocked from 20 to 30 hpf and allowed to recover until 36 hpf, *satb2* expression is still greatly reduced. Palatal precursors fail to express *satb2* (arrowhead in E) and only ventromedial crest in arches 1 & 2 express *satb2* (arrows in E & F). Later inhibition of Bmp signaling does not alter *satb2* expression. (G–J) 42 hpf embryos labelled with riboprobe to *satb2* in lateral (G, I) and ventral (H, J) views. In both control embryos (G, H) and embryos treated with dorsomorphin from 30 to 42 hpf (I, J), *satb2* is strongly expressed by the palatal precursors and in the ventral pharyngeal arches. l, lateral; m, medial; p, palatal precursors.

Ventral arch neural crest cells respond to Bmp signaling beginning by at least 24 hpf [18]. Therefore, we first tested if this initial Bmp response was essential for *satb2* expression. Embryos treated with dorsomorphin from 20 to 36 hpf showed a dramatic reduction in the level of *satb2* expression relative to untreated control embryos (Fig. 5 A–D). Compared to controls, expression of *satb2* in the palatal precursors was undetectable (Fig. 5 A & C, arrowhead) and only a small region of ventral medial second arch crest maintained a low level of *satb2* expression (Fig. 5 B & D, arrows). Even when dorsomorphin was washed off at 30 hpf and the embryos were allowed to further develop to 36 hpf, the expression of *satb2* expression was still dramatically reduced (Fig. 5 E & F), although not to the extent seen with Bmp blockage from 20–36 hpf. These analyses were performed in *BRE:mKO2* transgenics, allowing us to verify that Bmp signaling had been partially, albeit not completely, restored in the ventral neural crest (Figure S1). While the expression of *satb2* in the palatal precursors was still absent (Fig. 5 E, arrowhead), a small number of ventral medial crest cells within the first and second arch expressed *satb2* weakly (Fig. 5 F, arrows). While the partial recovery of *satb2* expression and the BRE response correlate with one another, blocking Bmp signaling from 30 to 42 hpf caused no gross alteration to *satb2* expression (Fig. 5 G–J). Collectively, these results demonstrate that the Bmp signal necessary for *satb2* expression is required predominantly prior to the initiation of *satb2* and that, following this initial signaling event, canonical Bmp signaling is dispensable for *satb2* expression.

**Figure 6:**
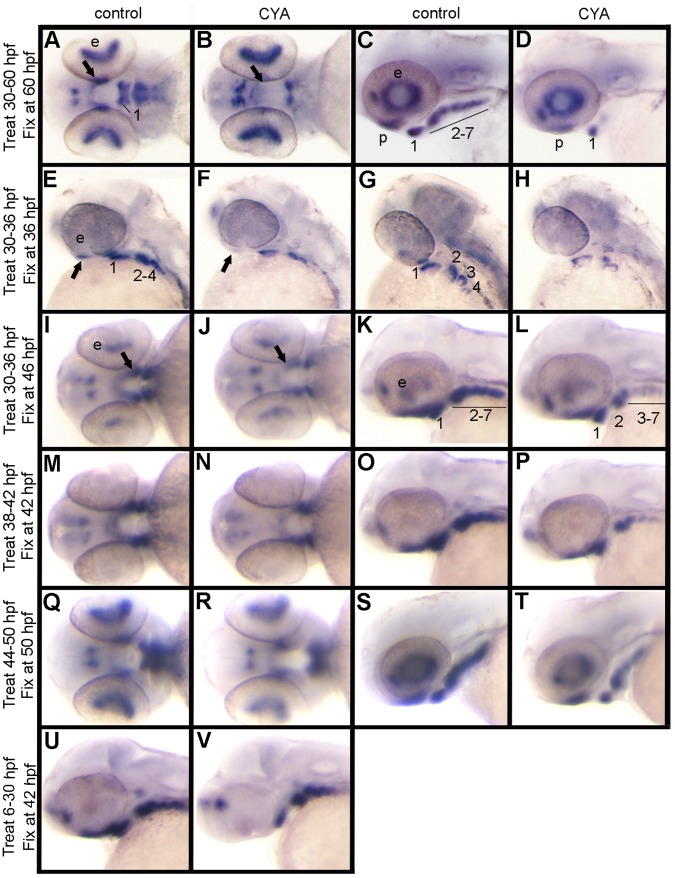
Continuous Hh signaling is required for *satb2* expression. Ventral (A, B, I, J, M, N, Q, R), lateral (C, D, E, F, K, L, O, P, S, T, U, V) and dorsal-lateral (G, H) images of *satb2* expression by *in situ* hybridization are shown in control and cyclopmaine-treated (CYA) embryos. (A, C) In controls at 60 hpf, *satb2* is expressed in palatal precursors and in the ventral region of each arch. (B, D) Embryos treated with cyclopamine from 30–60 hpf show a dramatic reduction of *satb2* expression in the palatal precursors, pharyngeal arch 1 and 2 as well as complete loss of expression in the posterior arches. (E–H) Compared to controls, embryos treated with cyclopamine from 30 to 36 hpf also show reduction of *satb2* expression in the palatal precursors and arches 1 and 2, with a more complete loss of expression in the more posterior arches. (I–L) If, however, embryos are removed from cyclopamine at 36 hpf and allowed to develop for 10 hours, there is a partial recovery of *satb2* expression. (M–T) Cyclopamine treatment either from 38 to 42 hpf (M–P) or 44 to 50 hpf (Q–T) results in mild reduction of *satb2* expression in the palatal precursors and pharyngeal arches. (U, V) While the maxillary domain is lost in embryos treated with cyclopamine from 6–30 hpf the expression in the ventral arches appears largely intact. CYA, cyclopamine; e, eye; b, brain; p, palatal precursors; pharyngeal arches are numbered in some panels for clarity.

Initiation of *satb2* expression in the pharyngeal arches shortly follows the initiation of *shha* expression in the pharyngeal endoderm, suggesting that Hh signaling may be more proximal to the initiation of *satb2* than Bmp signaling. Overall, we found that cyclopamine treatments that started at 30 hpf, just prior to the initiation of *satb2* expression, caused dramatic loss in *satb2* expression (Fig. 6 A–L). By blocking Hh between 30–60 hpf, corresponding with the apparent peak of *satb2* expression, we see the most dramatic reduction of *satb2* (Fig. 6 A–D). In these embryos only ventral arch 1 expressed *satb2* strongly, with weaker expression seen in the palatal precursors and ventral arch 2 (Fig. 6 C & D). Cyclopamine treatment from 30–36 hpf gave similar results (Fig. 6 E–H). If, however, embryos were treated between 30 to 36 hpf, rinsed and then reared for another 10 hours, partial recovery of *satb2* expression in the arches was observed (Fig. 6 I–L), suggesting that Hh signaling continues to be important for *satb2* expression. We performed further cyclopamine treatments to investigate if *satb2* requires a continuous Hh signal to maintain expression.

We used either a 4 or 6-hour treatment of cyclopamine to determine the temporal requirements for Hh signaling in *satb2* expression. Embryos were treated with cyclopamine between 38–42 hpf and 44–50 hpf and *satb2* expression assessed immediately following treatment (Fig. 6 M–P and Q–T, respectively). At both time points the cyclopamine-treated embryos showed reduction of *satb2* expression in the arches and subtle reduction in the palatal precursors. These observations suggest that continued Hh signaling is necessary to maintain an appropriate level of *satb2* expression.

During zebrafish palatogenesis, an early Hh signal from the presumptive ventral brain signals to the oral ectoderm 12 hours prior to the arrival of CNCC in the maxillary domain [8]. This early signal is required for CNCC condensation and proper palatogenesis to occur [8]. Thus, we asked whether or not an early Hh signal was necessary to ‘prime’ the ventral arch CNCC to express *satb2* later in development. We blocked Hh with cyclopamine between 6 hpf and 30 hpf then analyzed *satb2* expression at 42 hpf, once Hh signaling had recovered. Inhibiting Hh signaling during this time window disrupts the condensation of palatal precursors [8], preventing an analysis of this cell population. However, the level of *satb2* expression in the arches of treated embryos appeared similar to non-treated controls suggesting that an early Hh signal is not required to pre-pattern the ventral arch to express *satb2* (Fig. 6 U, V). Collectively, our inhibitor studies strongly suggest that Bmp signaling to the ventral pharyngeal arch mesenchyme is required prior to Hh signaling and that Hh signaling continues to be important for *satb2* throughout arch development.

## Discussion/Conclusions

### satb2 Expression Depends upon the Integration of Bmp and Hh Signaling

**Figure 7:**
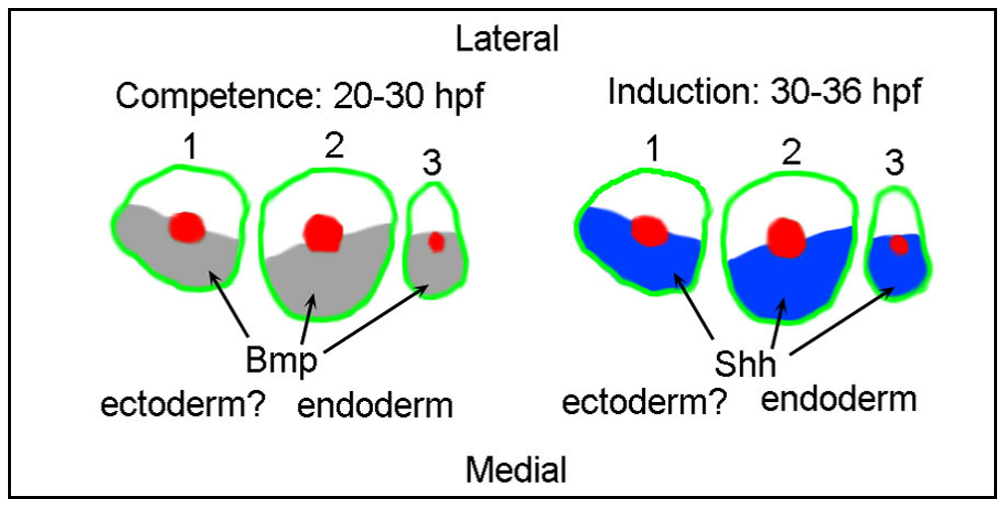
Model. Bmp signaling and Shh signaling are each necessary for proper *satb2* expression. Bmp signaling is necessary prior to both the induction of *satb2* and the time window when Hh signaling is necessary for *satb2* expression. Shh signaling is required immediately prior to and overlapping with the induction of *satb2* expression in the pharyngeal arches. Additionally, the induction of *satb2* occurs in crest in close proximity to *shha*-expressing endoderm. These findings support a model in which the early Bmp signal establishes competence of the neural crest to respond to the later Shh signal. The first three arches are shown in schematic and numbered.

We show that Hh and Bmp signaling are required for *satb2* expression in the developing pharyngeal arches (Fig. 7). The expression of *satb2* in both the maxillary domain and ventral pharyngeal arches require Bmp and Hh signaling. Blocking Bmp signaling from 20–36 hpf vastly decreases *satb2* expression in both the maxillary domain and ventral pharyngeal arches. Likewise, both maxillary domain and ventral arch expression of *satb2* is greatly reduced by blocking Hh signaling from 30–36 hpf. While these results show that the timing of Bmp and Hh signaling important for *satb2* expression is similar, it is possible that the precise mechanisms by which Bmp and Hh signals regulate *satb2* expression across neural crest domains differs. Future experiments aimed at characterizing the induction of *satb2* expression will be necessary to fully understand these potential differences.

The frequent coincident expression of Hh and Bmp ligands has long suggested functional interactions between these pathways [55]. During the development of numerous craniofacial elements these two signaling pathways have been shown to interact. Typically, these signaling pathways regulate one another through epithelial-mesenchymal interactions, such as during development of the palatal skeleton [26], [56], tooth germ [57], [58], frontonasal ectodermal zone [59] and scleral ossicles [60]. Additionally, ligands for both pathways have clear roles in the facial epithelia [56], [61]. While our data do not fully determine the sources of Shh and Bmp necessary for the induction of *satb2* expression, our analyses of *sox32* mutants show that the pharyngeal endoderm is clearly important for ventral expression in arches 2–7. It is likely that ectodermal expression of these ligands [8], [12], 53 is important for *satb2* expression in the ventral first arch.

It has been shown in zebrafish that ventral specification of the pharyngeal arches requires the Bmp and Edn1 pathways acting in opposition to dorsal Jagged/Notch signaling [16], [18], [19], [22]. Due to their known roles in other vertebrate species, these interactions are likely to be conserved in amniotes [13], [20], [62], [63], [64], [65]. The reception of these signals is necessary in the neural crest for proper specification [19], [22], [52], [66], [67], although Edn1 signaling to the facial epithelia is also important [68]. Appropriate specification of the pharyngeal arches also requires the reception of Hh signaling by the neural crest [11], [29]. Here, we show that neural crest cells must receive both Bmp and Hh signaling for the ventral expression of *satb2*. Hand2 is downstream of Edn1 and Bmp signaling during craniofacial development [14], [16], [18], [19] and is also necessary for *satb2* expression, at least in the second and more posterior arches. Our previous analyses have shown that *hand2* expression is retained in zebrafish *smo* mutants [11], therefore, we can conclude that Hand2 is necessary but not sufficient for appropriate *satb2* expression.

Because *hand2* is an Edn1 target [14], [16], it was surprising to find that *satb2* expression was normal in *edn1* mutants. However, *hand2* expression is not completely lost in zebrafish *edn1* mutants [16]. Thus, this residual *hand2* expression may activate *satb2* expression. Alternatively, expression of *satb2* may be influenced by an Edn1-independent effecter such as Mef2ca [66]. While *mef2ca* expression in the zebrafish pharyngeal arches does not require Edn1 signaling, the expression of Edn1 target genes requires Mef2ca function [66]. Differential regulation of *satb2* expression by Edn1 and Hand2 would not be completely surprising as it has been suggested that Hand2 maybe a branch point of Edn1 regulation during arch development [13].

While Bmp and Hh signaling are crucial for *satb2* expression, the timing of these signaling events would appear to be somewhat, although not completely, distinct. Even though the ventral pharyngeal arch continues to respond to Bmp signaling until at least 48 hpf [18], our dorsomorphin analysis suggests that signaling past 30 hpf is dispensable for *satb2* expression. In contrast, our cyclopamine data suggest that Hh signaling from 30 hpf onward is essential for appropriate *satb2* expression. In our cyclopamine treatments we never eliminated *satb2* expression, suggesting an incomplete inhibition of the Hh pathway or that some earlier Hh signaling is important in the expression of *satb2* in the ventral first and second arch. Even though the necessary Bmp signaling event precedes the initiation of *satb2*, it has recently been shown that Smad1/5 binds chromatin approximately 1 kb 5′ of *Satb2* in mouse mandibles [69]. While similar analyses of Gli binding regions near *Satb2* in neural crest have not been performed, a strong Gli3 binding region is located just over 100 kb 5′ of *Satb2* in neural tissue [70]. Because both Bmp and Hh signaling are necessary for *satb2* expression, we propose a model to be tested in which the earlier Bmp signal establishes competence for neural crest cells to respond to a later Hh signal (Fig. 7).

## Supporting Information

Figure S1 **Wash out of Dorsomorphin causes a partial restoration of Bmp signaling.** (A) Untreated 30 hpf *BRE:mKO2* embryos have rhobust transgene expression in the ventral pharyngeal arches (arrows). (B) Nearly all expression, except for some in the dorsal retina is lost following dorsomorphin treatment from 20–30 hpf. (C) At 36 hpf the expression of the *BRE:mKO2* transgene closely resembles that observed at 30 hpf. (D) Embryos treated with dorsomorphin from 20–30 hpf and then washed out of the drug show a partial recovery of *BRE:mKO2* expression in the ventral pharyngeal arches (arrows).
(TIF)
