# Supplementary figures and images for: Bmp and Shh Signaling Mediate the Expression of satb2 in the Pharyngeal Arches
Source: PLoS One. 2013 Mar 21;8(3):e59533. doi: 10.1371/journal.pone.0059533 (PMC3605343; doi:10.1371/journal.pone.0059533)

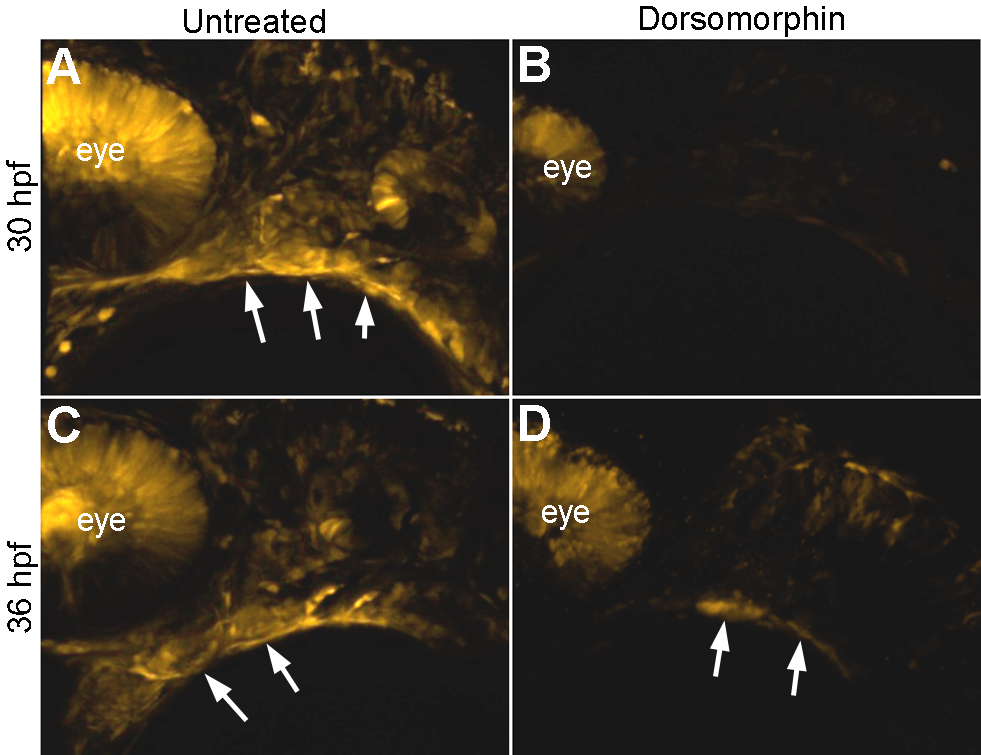

Supplement: Figure S1 — Wash out of Dorsomorphin causes a partial restoration of Bmp signaling. (A) Untreated 30 hpf BRE:mKO2 embryos have rhobust transgene expression in the ventral pharyngeal arches (arrows). (B) Nearly all expression, except for some in the dorsal retina is lost following dorsomorphin treatment from 20–30 hpf. (C) At 36 hpf the expression of the BRE:mKO2 transgene closely resembles that observed at 30 hpf. (D) Embryos treated with dorsomorphin from 20–30 hpf and then washed out of the drug show a partial recovery of BRE:mKO2 expression in the ventral pharyngeal arches (arrows). (TIF) [file pone.0059533.s001.tif]
